# Infarto agudo de miocardio sin enfermedad coronaria obstructiva secundario a tirotoxicosis por bocio multinodular tóxico

**DOI:** 10.47487/apcyccv.v1i4.90

**Published:** 2020-12-31

**Authors:** Harvey Julián Mejía Sandoval, Jorge Mario Palmezano Díaz

**Affiliations:** 1 Universidad Industrial de Santander. Departamento de Medicina Interna. Grupo GERMINA. Hospital Universitario de Santander. Bucaramanga, Colombia. Universidad Industrial de Santander Universidad Industrial de Santander Departamento de Medicina Interna Grupo GERMINA Bucaramanga Colombia; 2 Universidad Pontificia Bolivariana. Hospital Universitario de Santander. Bucaramanga, Colombia Universidad Pontificia Bolivariana Universidad Pontificia Bolivariana Hospital Universitario de Santander Bucaramanga Colombia

**Keywords:** Dolor torácico, Infarto de miocardio, Tirotoxicosis, Isquemia miocárdica, Chest pain, Acute myocardial infarction, Thyrotoxicosis, Myocardial ischemia

## Abstract

El infarto agudo de miocardio en ausencia de enfermedad coronaria obstructiva (MINOCA) representa un reto diagnóstico en la práctica clínica. En su abordaje diagnóstico se incluye la documentación de un sustrato isquémico, con un tratamiento que debe ser específico según la etiología identificada. Un grupo de estos pacientes presenta isquemia secundaria a una alteración en la relación aporte/demanda de oxígeno. Se presenta el caso de una paciente de sexo femenino y mediana edad, con hipertiroidismo severo y tirotoxicosis, que debuta con un síndrome coronario agudo, con troponina T marcadamente elevada y arterias coronarias sin lesiones significativas en la coronariografía. El tratamiento fue dirigido a manejar la causa desencadenante, de manera inicialmente farmacológica y posteriormente quirúrgica, con resolución total del cuadro, mejoría sintomática de la paciente y disminución de los biomarcadores cardíacos.

## Abstract (en)

Acute myocardial infarction in the absence of obstructive coronary artery disease (MINOCA) represents a diagnostic challenge in clinical practice. Documentation of an ischemic substrate is necessary for its diagnostic approach, with a treatment that must be specific according to the identified etiology. A group of these patients presents ischemia secondary to an alteration in the oxygen supply/demand ratio. We present the case of a female adult patient, with severe hyperthyroidism and thyrotoxicosis, who develops an acute coronary syndrome, with elevated troponin T and normal coronary arteries on coronariography. Treatment was aimed at treating the triggering cause, initially pharmacologically and later surgically, with a total resolution of the condition, symptomatic improvement in the patient, and a decrease in cardiac biomarkers.

El infarto agudo de miocardio en ausencia de enfermedad coronaria obstructiva (MINOCA), representa una situación especial con una amplia variedad de posibles etiologías. Para su diagnóstico debe cumplirse con la cuarta definición universal de infarto de miocardio, descartar la presencia de enfermedad coronaria obstructiva (definida como aquella con lesiones obstructivas mayores o iguales al 50%, en un vaso mayor epicárdico) y la ausencia de un diagnóstico alternativo para la presentación clínica ^1^.

El perfil clínico y demográfico de estos pacientes difiere sustancialmente de otros tipos de infarto, caracterizándose por ser pacientes más jóvenes y con una representación importante del sexo femenino, hasta en un 50% de los casos. También se ha descrito una menor proporción de elevación del segmento ST en el electrocardiograma y niveles más bajos de troponinas, respecto a los pacientes con enfermedad obstructiva ^2^. Puede clasificarse según la presencia o ausencia de causa ateroesclerótica ^1^.

El hipertiroidismo como origen de este cuadro ha sido esporádicamente descrito, con diferentes mecanismos fisiopatológicos propuestos. Presentamos el caso de un paciente con diagnóstico de hipertiroidismo severo con tirotoxicosis, quien se presenta con un síndrome coronario agudo, sin enfermedad coronaria obstructiva.

## Reporte de caso

Paciente de sexo femenino de 49 años, con cuadro clínico de ocho horas de evolución, caracterizado por dolor precordial súbito, que inicia en reposo, opresivo, irradiado a cuello, de intensidad severa (9/10 en la escala análoga del dolor), asociado a diaforesis, náuseas y emesis de contenido alimentario. Como antecedentes se destacan la presencia de hipertensión arterial, diagnosticada hace cinco meses, en manejo farmacológico con enalapril y la presencia de un nódulo tiroideo en estudio, sin diagnóstico previo de hipertiroidismo. Sin antecedentes familiares de enfermedad cardiovascular. La paciente no refirió otros síntomas a la revisión por sistemas.

**Figura 1:**
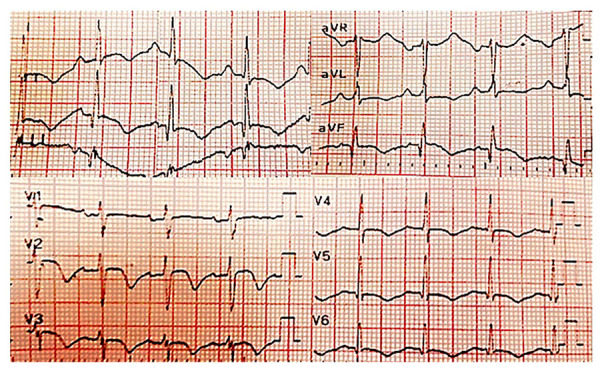
Electrocardiograma de ingreso. Se evidencia taquicardia sinusal, prolongación del intervalo QT corregido (530 milisegundos, por el método de Bazett) e inversión asimétrica de la onda T en derivaciones V2-V6.

**Figura 2:**
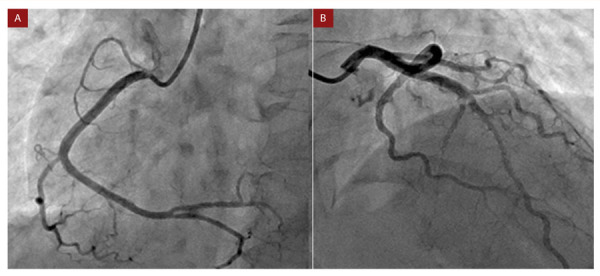
Arteriografía coronaria. A. Se observa arteria coronaria derecha dominante, con sus ramificaciones, sin lesiones obstructivas significativas. B. Arteria coronaria izquierda y sus ramas, sin evidencia de lesiones obstructivas.

Al examen físico de ingreso, presentaba una tensión arterial de 120/70mmHg; frecuencia cardiaca de 120 latidos por minuto; frecuencia respiratoria de 18 por minuto; temperatura 36,6 °C, y saturación 96% al ambiente. Se encontró una tiroides palpable, de consistencia nodular. No se auscultaron soplos en cuello ni en el ámbito cardiopulmonar. No se evidenciaron otras alteraciones. El electrocardiograma de ingreso reportó la presencia de taquicardia sinusal, un intervalo QT corregido prolongado (530 milisegundos), junto con inversión asimétrica de la onda T en derivaciones V2 - V6 **(**Figura 1**)**. Se realizaron biomarcadores cardiacos con franca elevación de la troponina T ultrasensible (1864,5 ng/L, valor normal: 0-2 ng/L), considerándose un infarto agudo de miocardio sin elevación del segmento ST. Se inició tratamiento con ácido acetil salicílico 300 mg, clopidogrel 300 mg, enoxaparina 50 mg cada doce horas, y se realizó arteriografía coronaria de urgencia, con documentación de arterias coronarias epicardicas sin enfermedad obstructiva significativa **(**Figura 2**)**. La ventriculografía y el ecocardiograma transtorácico no mostraron trastornos segmentarios de la contractilidad, y se encontró una fracción de eyección del ventrículo izquierdo, del 65%.

Dentro de los estudios complementarios se realizó análisis de la función tiroidea, con evidencia de hipertiroidismo severo (TSH: menor a 0,05 uUI/mL, valor normal: 0,25 -5 uUI/mL; T4 libre: 100 pmol/L, valor normal: 12 - 22 pmol/L). La escala de Burch Wartofsky se calculó en 25 puntos estableciéndose riesgo inminente de tormenta tiroidea. No hubo alteraciones en la función renal o hepática **(**Tabla 1**)**.

**Tabla 1:** Pruebas de laboratorio realizadas

Día	Prueba de laboratorio	Resultado
1	TSH	<0,05 u Ul/ mL - VR (0,25 - 5 uUl/ mL)
1	Tiroxina libre	100 pmol/L - VR (12 - 22 pmol/L)
1	Troponina T	1864 ng/L - VR (0 - 2 ng/L)
1	Creatinina	0,46 mg / dL
1	ASAT	32,1 U/L - VR (6 -32 U/L)
1	ALAT	40,10 U/L - VR (6 - 33 U/L)
1	Bilirrubina total	0,64 mg/ dL (0,2 - 0,9 mg/dL)
5	Troponina T control	196 ng/L - VR (0 - 2 ng/L)
8	Tiroxina libre control	40,5 pmol/L - VR (12 - 22 pmol/L)
32	Tiroxina libre control	13,6 pmol/L - VR (12 - 22 pmol/L)

TSH: hormona estimulante de tiroides; ASAT: aspartato amino transferasa; ALAT: alanino amino transferasa; VR: valor de referencia

**Figura 3:**
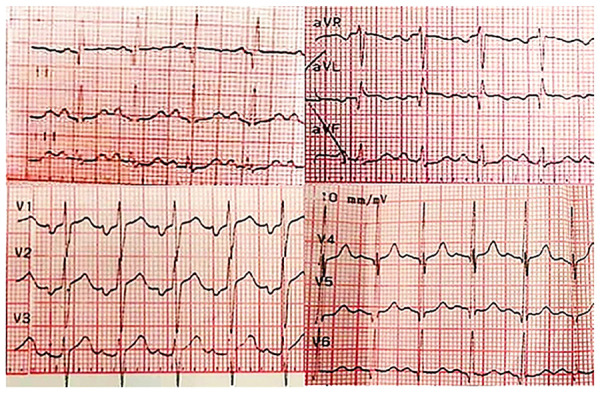
Electrocardiograma de control (cinco días después). Se observa taquicardia sinusal; presencia de ondas Q en las derivaciones I a y aVL; normalización del intervalo QT corregido (420 milisegundos [método de Bazett]) y desaparición de los cambios en la onda T, previamente observados.

Se inició manejo con metimazol, lugol, propranol, colestiramina e hidrocortisona, obteniéndose mejoría clínica, con resolución de los síntomas referidos por la paciente en los primeros cinco días, y paraclínica con descenso de la tiroxina libre en el control realizado a la semana de tratamiento. En el electrocardiograma de control a los cinco días, se observó la presencia de ondas Q en las derivaciones I y aVL (sugiriendo el establecimiento de necrosis debido a la lesión causada), normalización del intervalo QT corregido (420 milisegundos) y desaparición de los hallazgos en la onda T evidenciados en el electrocardiograma de ingreso **(**Figura 3**)**.

Se diagnosticó un infarto agudo de miocardio sin enfermedad coronaria obstructiva (tipo 2), secundario a un desbalance entre aporte y demanda de oxígeno, desencadenado por tirotoxicosis. El tratamiento con ácido acetil salicílico, clopidogrel y enoxaparina, fue suspendido una vez obtenido el reporte de la coronariografía.

Para el estudio del hipertiroidismo se realizó una ecografía de tiroides que mostró un aumento del volumen de la glándula con ecogenicidad heterogénea, el lóbulo derecho midió 50x28x27 mm y el izquierdo 55x28x23 mm, los hallazgos indicaron bocio multinodular tóxico. Los profesionales médicos del Servicio de Endocrinología y Cirugía de Cabeza y Cuello decidieron realizar una tiroidectomía total, la cual se efectuó sin complicaciones.

El reporte histopatológico de la muestra quirúrgica concluyó bocio multinodular, negativa para malignidad y sin evidencia de tejido paratiroideo. La citología mostró hallazgos benignos, Bethesda categoría II. La paciente presentó una evolución satisfactoría, con resolución del hipertiroidismo a nivel paraclínico y sin recurrencia del dolor precordial; luego se indicó reemplazo hormonal permanente con levotiroxina.

## Discusión

La presencia de un síndrome coronario agudo en un paciente adulto de mediana edad, con riesgo cardiovascular bajo, debe encender las alarmas y dirigir la búsqueda a causas menos convencionales. En el caso presentado, la elevación marcada de la troponina T ultrasensible, junto con la sintomatología, confirmaron la presencia de un infarto de miocardio en tanto que la arteriografía coronaria sin enfermedad obstructiva significativa trasladó el caso a un escenario particular.

El MINOCA ocurre en 5 a 6% de los casos de infarto de miocardio sometidos a coronariografía ^3^. Su definición actualizada, dentro del marco de la cuarta definición universal de infarto de miocardio, exige la presencia de un contexto isquémico, para diferenciarlo de otros cuadros de lesión miocárdica ^1^.

El estado de hipertiroidismo severo con tirotoxicosis, produce importantes cambios hemodinámicos a nivel cardiovascular, incluyendo aumento en la frecuencia cardiaca, volumen latido, contractilidad miocárdica y fracción de eyección; esto es similar a lo que ocurre en un estado hiperadrenérgico. Si bien no existe un aumento de las catecolaminas séricas, sí se presenta una mayor densidad de receptores beta adrenérgicos, lo cual genera el incremento de la sensibilidad a las catecolaminas. Esto, finalmente, ocasiona un mayor consumo de oxígeno a nivel tisular ^2^^,^^4^.

En algunos pacientes esta situación puede predisponer a un proceso de isquemia e inclusive infarto del miocardio, lo cual es más frecuente en presencia de aterosclerosis de base. En estos casos, se favorece la ruptura de una placa ateroesclerótica, ocurre embolización distal y, posteriormente, lisis del trombo, evidenciando entonces coronarias sin obstrucción significativa (origen ateroesclerótico del MINOCA) ^5^. Otros mecanismos propuestos incluyen la presencia de vasoespasmo coronario por un desequilibrio en la inervación autonómica cardiaca, junto con alteración en las concentraciones de las prostaglandinas ^6^.

En el caso expuesto no se documentó ateroesclerosis y las arterias coronarias no evidenciaron ningún grado de obstrucción en la angiografía; esta es una situación muy llamativa y esporádica en la práctica clínica y, bajo este sustrato, se clasificó como un infarto tipo 2, correspondiente al subgrupo de MINOCA de origen no ateroesclerótico, ocasionado por alteración en la relación aporte - demanda de oxígeno ^1^^,^^7^.

El tratamiento de elección en estos casos sigue siendo discutido, con escasa literatura basada en la evidencia. La Asociación Americana del Corazón (AHA) sugiere un manejo individualizado, teniendo como fundamento la identificación y corrección de la etiología subyacente ^8^. En este reporte de caso, el manejo específico inicial fue realizado con fármacos antitiroideos, con mejoría total de la sintomatología de la paciente, resolución de los cambios electrocardiográficos y disminución notoria de los biomarcadores cardíacos. El tratamiento definitivo se hizo quirúrgicamente, con tiroidectomía total, dada las dimensiones de la glándula y la necesidad de una corrección pronta del hipertiroidismo, según las recomendaciones de la Asociación Americana de Tiroides (ATA) ^9^, e identificando como causa desencadenante un bocio multinodular tóxico, con confirmación histopatológica. La buena evolución de la paciente en el tiempo, resalta la importancia de identificar a tiempo la causa en estos casos y dar un tratamiento acorde con los hallazgos. 

## Conclusiones

El infarto agudo de miocardio en ausencia de enfermedad coronaria obstructiva, representa un reto diagnóstico y terapéutico para el clínico. Su manejo debe ser individualizado y el abordaje integral según la causa identificada. La presencia o ausencia de enfermedad ateroesclerótica determinará la necesidad de otras terapias adicionales. Así mismo, cuando la fisiopatología corresponda a un desbalance aporte - demanda de oxígeno, el objetivo terapéutico intuitivo debe ser revertir la condición causal. Se debe sospechar y evaluar la presencia de enfermedad tiroidea en pacientes jóvenes de bajo riesgo cardiovascular, donde el hipertiroidismo cumple una función esporádica, pero de gran relevancia.
